# Whole-Genome Sequences of Two Beak and Feather Disease Viruses in the Endangered Swift Parrot (*Lathamus discolor*)

**DOI:** 10.1128/genomeA.00842-13

**Published:** 2013-11-27

**Authors:** Subir Sarker, Seyed A. Ghorashi, Jade K. Forwood, Shane R. Raidal

**Affiliations:** School of Animal and Veterinary Sciences, Charles Sturt University, Wagga Wagga, New South Wales, Australiaa; School of Biomedical Sciences, Charles Sturt University, Wagga Wagga, New South Wales, Australiab; Graham Centre for Agricultural Innovation, Wagga Wagga, New South Wales, Australiac

## Abstract

Two complete genomes of beak and feather disease virus (BFDV) were characterized from *Lathamus discolor*, the Australian swift parrot. This is the first report of BFDV complete genome sequences in this host. The completed BFDV genomes consist of 1,984 nucleotides encoding two open reading frames with 99.7% pairwise nucleotide identity.

## GENOME ANNOUNCEMENT

Psittacine beak and feather disease (PBFD) is a well-recognized viral threat to a wide variety of psittacine bird species globally, and it typically causes immunosuppression and chronic symmetrical feather loss, as well as beak and claw deformities (1–4). The etiological agent of the disease, beak and feather disease virus (BFDV), composed of a compact circular ambisense single-stranded DNA (ssDNA) genome of approximately 2,000 nucleotides encoding a replicase (Rep) and a single capsid protein (Cap) (2, 5–7), is capable of infecting all *Psittaciformes* since it has been reported in >60 species of cockatoos and parrots (8–10). In the present study, we report two complete genomes of BFDVs from the endangered swift parrot (*Lathamus discolor*) (http://www.iucnredlist.org/details/106001485/0).

The BFDV viral genomes were amplified from dried blood spots collected from two wild swift parrots (year of sampling, 2004; GPS location, -43.407043°S 147.322540°E), and the genomic DNA was extracted using established protocols (11–13). To amplify the entire viral genome, a published primer (BFDV-P2, 5′-AACCCTACAGACGGCGAG-3′) (11) and designed primers (BFDV-J-R, 5′-TTGGGTCCTCCTTGTAGTGG-3′; BFDV-I-F, 5′-GCAAACTGACGGAATTGAACATA-3′; and BFDV-C-R, 5′-CGTCCAACGATGGCATAGT-3′) were used. The reactions for different sets of primers were optimized, and the optimized reaction mixture contained 3 µl extracted genomic DNA, 2.5 µl of 10× High Fidelity PCR buffer (Invitrogen), 1 µl of 25 µM each primer, 1 µl of 50 mM MgSO_4_, 4 µl of 1.25 mM each deoxynucleoside triphosphate (dNTP), 1 U Platinum *Taq* DNA polymerase High Fidelity (Invitrogen), and distilled water (dH_2_O) added for a final volume of 25 µl. The optimized PCR conditions were as follows: 95°C for 3 min, followed by 40 cycles of 95°C for 30 s, 57°C for 45 s, and 68°C for 2 min, and finally 68°C for 5 min. The extension time for the second set of primers (BFDV-I-F and BFDV-C-R) was 1.5 min instead of 2 min. The amplified PCR products were TA cloned into pGEM-T vector (Promega) and sequenced at the Australian Genome Research Facility (AGRF) Ltd. (Brisbane, Australia). The sequence contigs were assembled, and the entire BFDV genomes were constructed using the Geneious software.

Two newly amplified BFDV genomes (GenBank accession no. KF673335 and KF673336), along with all other BFDV genome sequences from GenBank, were aligned using the MAFFT L-INS-i algorithm (14); they exhibit 99.7% pairwise nucleotide identity with each other and >87.0% nucleotide sequence homology with other BFDV genomes.

While habitat loss was considered to be the major threat to swift parrots, the spread of infectious diseases, especially PBFD, was also highlighted as a key threat (12). Therefore, the complete genome sequences of BFDVs for the first time may provide novel insights into the viral evolutionary history in this host species.

### Nucleotide sequence accession numbers.

The complete genome sequences of the two BFDVs were deposited at GenBank under accession no. KF673335 and KF673336.
